# Interaction between alveolar macrophages and epithelial cells during *Mycoplasma pneumoniae* infection

**DOI:** 10.3389/fcimb.2023.1052020

**Published:** 2023-04-11

**Authors:** Yazhi Xue, Mengyao Wang, Hongbing Han

**Affiliations:** ^1^ Beijing Key Laboratory of Animal Genetic Improvement, College of Animal Science and Technology, China Agricultural University, Beijing, China; ^2^ Institute of Thoracic Oncology and Department of Thoracic Surgery, West China Hospital of Sichuan University, Chengdu, China

**Keywords:** *Mycoplasma pneumoniae*, epithelial cells, alveolar macrophages, cytokines, extracellular vesicles

## Abstract

*Mycoplasma pneumoniae*, as one of the most common pathogens, usually causes upper respiratory tract infections and pneumonia in humans and animals. It accounts for 10% to 40% of community-acquired pneumonia in children. The alveolar epithelial cells (AECs) are the first barrier against pathogen infections, triggering innate immune responses by recruiting and activating immune cells when pathogens invade into the lung. Alveolar macrophages (AMs) are the most plentiful innate immune cells in the lung, and are the first to initiate immune responses with pathogens invasion. The cross-talk between the alveolar epithelium and macrophages is necessary to maintain physiological homeostasis and to eradicate invaded pathogen by regulating immune responses during *Mycoplasma pneumoniae* infections. This review summarizes the communications between alveolar macrophages and epithelial cells during *Mycoplasma pneumoniae* infections, including cytokines-medicated communications, signal transduction by extracellular vesicles, surfactant associated proteins-medicated signal transmission and establishment of intercellular gap junction channels.

## Introduction

*Mycoplasma pneumoniae* pneumonia is an acute respiratory infection disease caused by *Mycoplasma pneumoniae* (MP). Among the pathogens formerly known as “primary atypical pneumonia”, MP is the most common, accounting for 10-40% of community-acquired pneumonia cases. Recent studies have shown that younger animals and school-age children are susceptible to *Mycoplasma pneumoniae* pneumonia (Michelow et al., 2004; Defilippi et al., 2008; Kim et al., 2009). It can induce upper and lower respiratory tract infection in humans and animals and spread all over the world. Apart from respiratory disease, the organism also has the ability to produce a wide spectrum of non-pulmonary manifestations including hemolytic anemia, polyarthritis, erythema multiforme and the diseases in nervous system, liver and heart (Razin et al., 1998). Severe MP infections can also lead to death (Lee et al., 2018). It has been reported that the mortality rate of elderly patients with MP is as high as 30% (Khoury et al., 2016). MP, a gram-negative microorganism, is the smallest self-replicating bacterium with an extremely small genome (Chmura et al., 2008; Ledford et al., 2012). As a prokaryotic pathogen, it has three membranes without cell wall, and its survival depends on the exchange of nutrients with the host (Segovia et al., 2018). The pathogen are spread through direct contact between infected and susceptible people, as well as through droplets emitted by infected people when they sneeze, cough or speak (Hyde et al., 2001). After MP enters the respiratory tract with air, due to the lack of cell wall, the MP membrane can be in direct contact with epithelial cells, thus transferring or exchanging membrane components (Essandoh et al., 2016). MP can produce various pathogen associated molecular patterns (PAMPs), such as membrane lipoprotein, polysaccharide and invasive ribozyme, which can cause a series of pathophysiological changes of host (Somarajan et al., 2010; Daubenspeck et al., 2015; Shimizu, 2016). MP induces host cells to produce interleukin (IL)-8, tumor necrosis factor (TNF)-α and other pro-inflammatory cytokines. The content of IL-8 and TNF-α in patient’s serum increased with the aggravation of MP infection (Yu et al., 2018).

Pulmonary epithelium is the site for gas exchange between lung and blood, and is the first mucus barrier line for defense against foreign invasion and pathogenic factors. The mature alveolar epithelial cells (AECs) is composed of type I (AECIs) and type II (AECIIs) alveolar epithelial cells, accounting for 95% and 5%, respectively (Whitsett and Alenghat, 2015). AECIs are flat cells covering capillaries that provide the thin surface of the alveoli and are the most important site for gas exchange. In addition to serving as a physical barrier against pathogens and various entering environmental particles, AECIIs also are involved in the immune response, and maintain the balance of lung environment (Guillot et al., 2013). After entering the respiratory tract, MP adheres to AECIIs through surface adhesion molecules to induce host cells the production of TGF-β and extracellular vesicles carrying miRNA, which activate alveolar macrophages to clear MP.

**Figure 1:**
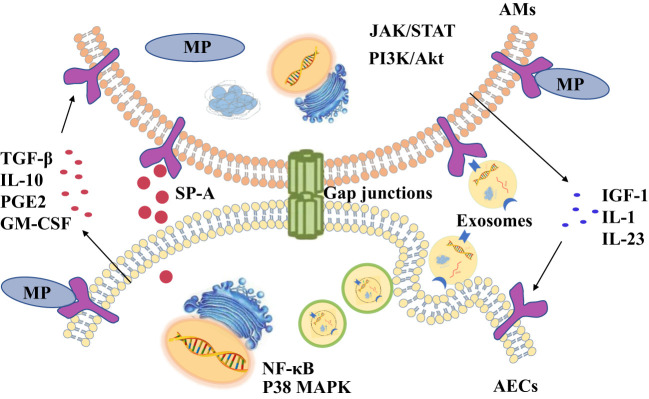
There are four ways of communication between alveolar macrophages (AMs) and alveolar epithelial cells (AECs) during *Mycoplasma pneumoniae* (MP) infections, including cytokines, exosomes, surfactant-associated protein A (SP-A) and gap junction channels.

Alveolar macrophages (AMs), free in the alveolar cavity, are primary immune cells in the lung, and are the key cellular sensors for pathogens with the characteristics of phagocytosis and secretion of cytokines (Malaviya et al., 2010; Byrne et al., 2015; Allard et al., 2018). The membrane lipoprotein of MP binding to Toll-like receptors (TLRs) on AMs activates signaling pathways such as nuclear factor (NF)-κB, and causes the secretion of pro-inflammatory cytokines, promoting the aggregation of neutrophils and phagocytosis of pathogens (Zhang et al., 2000). More and more evidence demonstrate that the interaction of structural cells and immune cells each other are essential to resist external pathogens (Yuan et al., 2014; Tran et al., 2017). In the lung, AMs and AECs communicate with each other to coordinate their actions to maintain pulmonary homeostasis and gas exchange (Tao and Kobzik, 2002). This paper focuses on summarizing the research on the signal communications between AECs and AMs during MP infection (**Figure 1**).

## Cytokines-medicated communications between AMS and AECS during MP infection

After contacting host cells, invasive pathogens are first recognized by cell surface pattern recognition receptors (PRRs), causing innate immune responses. One of the most characteristic PRRS groups is TLRs (Zarnke and Rosendal, 1989). By forming homodimers or heterodimers, TLRs can recognize the molecular pathogenic patterns of bacteria, viruses and fungi (Akira et al., 2001; Guo et al., 2018), and then induce immune responses, promoting the synthesis and release of various inflammatory cytokines and chemokines (Palm et al., 2015; Johnston and Corr, 2016). Existing studies have shown that the TLR2 and TLR4 on the cell surface of barrier cells and immune cells are necessary for recognizing MP, and they can initiate a series of downstream signaling responses, including the NF-κB, interferon (IFN) and inflammasome signaling pathways, leading to the production of corresponding pro-inflammatory or anti-inflammatory cytokines (Iwasaki and Medzhitov, 2004; Akira et al., 2006). The role of cytokines in pulmonary immune has been increasingly investigated in clinical animal and *in vitro* studies (Wang et al., 2020; Bai et al., 2021). When the lungs are stimulated, AECIIs and AMs can also communicate signals with each other through cytokines to regulate immune function and clear pathogens (Thorley et al., 2007; Kannan et al., 2009; Rana et al., 2020).

**Figure 2:**
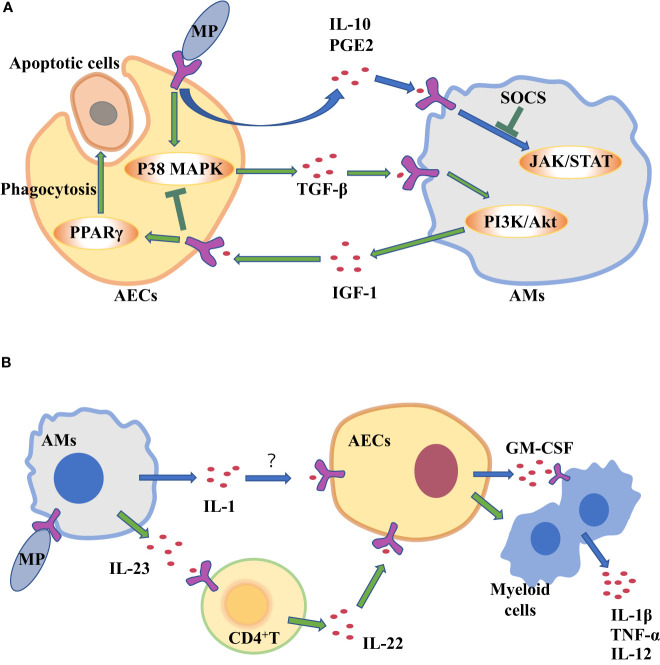
Alveolar macrophages (AMs) and alveolar epithelial cells (AECs) communicate with each other through cytokines to clear *Mycoplasma pneumoniae* (MP). **(A)** AECs attacked by MP secrete cytokines to regulate inflammation in AM. **(B)** Cytokine secretion by MP-challenged AMs amplifies inflammation in AECs.

AECIIs can secrete PGE2 and IL-10 that promote AMs to secrete SOCS protein, and SOCS protein can inhibit inflammatory STAT pathway (Bourdonnay et al., 2015). Previous studies have confirmed that serum IL-10 levels are higher in patients with mycoplasma pneumonia (Hassan et al., 2008; Salvatore et al., 2008). So AECIIs communicate with AMs through increasing anti-inflammatory factors such as IL-10 to prevent excessive lung inflammation from MP (**Figure 2A**). But compared with mild mycoplasma pneumonia, serum IL-10 levels in patients with severe mycoplasma pneumonia are significantly lower (Ding et al., 2016). The study found that MP can inhibit the secretion of anti-inflammatory factor IL-10 in AECIIs, regulating the inflammatory response in the lung (Shi et al., 2021).

The two-way communication between AECs and AMs can balance alveolar environment. After being stimulated by external stimuli, not only do AECs secrete cytokines to transmit signals to AMs, but AMs also act on AECs by cytokines. Antimicrobial immune responses of AMs are thought to be initiated by PRRs sensing PAMPs. Subsequently, there is the recruitment of myeloid cells, such as monocytes (MCS) and neutrophils, which amplifies the inflammatory response for controlling infection (Chaplin, 2010; Iwasaki and Medzhitov, 2015). However, many pathogens can infect and replicate within alveolar macrophages, which inhibits the translation in infected macrophages to escape the innate immune defense of macrophages (Copenhaver et al., 2014; Moss et al., 2019). As a result, the secretion of cytokines decreases, such as tumor necrosis factor (TNF) and interleukin-12 (IL-12) (Brieland et al., 1998; Copenhaver et al., 2015), which are needed to control infection (Brieland et al., 1998). Studies have shown that Mycoplasma ovipneumoniae can enter AMs and evade immune clearance by AMs (Gao et al., 2021). However, mycoplasma infection can induce to produce IL-1β in mouse or human macrophages *in vitro* (Quentmeier et al., 1990; Muhlradt and Schade, 1991; Herbelin et al., 1994; Kostyal et al., 1994). IL-1 induces the production of granulocyte-macrophage colony stimulating factor (GM-CSF) in alveolar epithelium (Liu et al., 2020). It’s true that the expression level of GM-CSF from AECs is increased in patients with MP (Xu et al., 2021), which promotes the production, proliferation and differentiation of granulocytes and monocytes, thereby amplifying the inflammatory response and promoting host defense (Roach et al., 2020). In a word, during MP stimulates AMs, increased IL-1 from AMs acts on epithelial cells, thereby up-regulating GM-CSF to amplify inflammation (**Figure 2B**).

CD4^+^ T cells serve as mediators in the cytokine-mediated communications between AMs and epithelial cells. It has been reported that IL-23 from AMs is increased in the response of MP infection (Iwakura and Ishigame, 2006, Wu et al., 2007a). IL-23 can induce CD4+T cells to secrete IL-22 which has a protective effect on epithelial cell barrier (Hoegl et al., 2011). IL-23 from macrophages act on CD4^+^ T cells to increase IL-22 (Bernshtein et al., 2019).That is to say, when MP attacks AMs, the up-regulating IL-23 secreted by AMs will act on CD4 ^+^ T cells, and then CD4 ^+^ T cells promote chemokine secretion from AECIIs, which recruits neutrophils and promote inflammatory response (**Figure 2B**). Although AECIIs have traditionally been considered distal lung cells in response to harmful environmental stimuli, in fact, some researchers have suggested that AECIs are a more important source of pro-inflammatory signals than AECIIs (Wong and Johnson, 2013). AECIs are thought to be more involved in pro-inflammatory responses, in part because they have a lot of surface contact with AMs that are present in the alveolar space. The role of AECIs in the inflammatory response induced by MP remains to be explored.

The persistence of inflammatory response is unfavorable to the body (Wang et al., 2017). A strong inflammatory response leads to local tissue damage, epithelial cell damage, and increased vascular leakage, all of which can lead to lung dysfunction and even death (Grommes and Soehnlein, 2011). At present, it is believed that most of the damage of MP to the body is caused by excessive immune response (Waites and Talkington, 2004). Sheep infected with MP are more likely to be infected by other microorganisms, so there may be immunosuppressive effect in the process of MP infection (Gao et al., 2021). It is well known that MP stimulates lung epithelial cells to produce transforming growth factor-β (TGF-β), which can cause apoptosis of AECs and promote pulmonary fibrosis (Lin et al., 2018). TGF-β from AECs can induce AMs to produce anti-inflammatory cytokines insulin-like growth factors (IGF-1) through PI3K/Akt signaling pathway. In turn, IGF-1 inhibits PAMPs-induced p38 MAPK activation and inflammatory cytokine production in AECs, and promote AECs to phagocytose apoptotic cells through PPARγ (Mu et al., 2020). In the mouse allergic asthma model, MP induces the production of IFN- γ through TLR2, which reduces the production of TGF- β 2 in bronchial epithelial cells and down-regulates the expression of airway mucin (Wu et al., 2007b). Therefore, MP stimulates TGF-β secreted by AECs. It can act on AMs to secrete IGF-1 to inhibit excessive lung inflammation (**Figure 2A**).

In general, when AMs are stimulated by MP, they recruit neutrophils and amplify inflammation by acting on AECs through pro-inflammatory cytokines. When MP enters the lungs and acts on AECs, epithelial cells will secrete IL-10 to inhibit macrophage inflammatory response, and TGF-β will also stimulate macrophage to secrete IGF-1 to inhibit epithelial inflammatory response.

## Cross-talk between AMS and AECS through exosomes during MP infection

The effective defense system of lung epithelium operates by releasing a variety of mediators, including cytokines, chemokines, nucleic acid molecules and antimicrobial peptides (Yamamoto et al., 2012). It was found that the swine epithelial NPTr cells infected with Mycoplasma hyopneumoniae were differentially expressed in microRNAs (miRNAs) related to antioxidant response and mRNA related to ciliary function. These differentially expressed genes were detected in the exosomes secreted by epithelial cells (Mucha et al., 2020). Exosomes, as an effective cellular signal and a communication system, is an important regulator of various pathophysiological conditions (Cho et al., Silverman and Reiner, 2011). Exosomes are extracellular vesicles (50-150 nm) that eukaryotic cells release under normal or pathological conditions (Rome, 2013). Extracellular vesicles in bronchoalveolar lavage fluids contain a large number of miRNAs, which contribute to innate immune responses after bacterial lung infection (Lee et al., 2019). In addition to miRNAs, extracellular vesicles can also transmit information between cells through proteins, lipids and nucleic acids. These proteins, lipids and nucleic acids seem to be randomly selected, and some specific molecular groups are preferentially packaged into vesicles (Schorey et al., 2015). Extracellular vesicles released by cells, have the potential to selectively interact with specific target cells and participate in intercellular communication (Rana et al., 2012; Forterre et al., 2014; Zhang W et al., 2018).

The results show that AMs can effectively absorb exosomes *via* tracheal perfusion, and exosomes siRNA or miRNA molecules are functional in modulating lung inflammation (Zhang D et al., 2018). At present, three methods have been observed for cell uptake of exosomes: namely endocytosis (Feng et al., 2010; Fitzner et al., 2011; Tian et al., 2014), receptor-mediated internalization (Svensson et al., 2013) and direct binding to target cell membranes (Tian et al., 2013). H-G Moon et al. induced human epithelial cells to produce extracellular vesicles by hyperoxia, and treatment of AMs with them resulted in increased secretion of pro-inflammatory cytokines and macrophage inflammatory protein 2 (MIP-2). In the lung tissue of mice treated with extracellular vesicles, the influx of macrophages and neutrophils increased significantly (Moon et al., 2015; Lee et al., 2016). Zunyong Feng et al. found that miR-27b-3p-carrying exosomes from AECIIs can induce AMs to produce pro-inflammatory signals. These exosomes play a regulatory role by regulating the expression of RGS1 in macrophages and then regulating the intracellular PLC-IP3R signal-dependent inflammatory response (Feng et al., 2021). It also have been confirmed that AECIs can produce a large number of miRNA-rich extracellular vesicles. And the miRNAs contained in these extracellular vesicles are actively delivered to AMs, thus promoting inflammasome activation, neutrophil recruitment and M1-macrophage polarization (Lee et al., 2019).

However, some studies have found that the release of exosomes can damage the host immune defense. Mycoplasma can destroy the mitochondrial DNA (mtDNA) in cells (Robberson et al., 1980). And the damaged mtDNA of AECs and AMs can be released in exosomes. The release of epithelial exosomes will destroy the phagocytosis of macrophages, and macrophages will also release exosomes to destroy the integrity of the alveolar epithelial barrier. That is to say, the release of damaged mtDNA-rich exosomes after mycoplasma stimulation can lead to the cross-talk of damage signals between AECs and AMs, impairing the host’s immune defense against respiratory infection (Sadikot et al., 2019). Moreover, mycoplasma can spread its components by Myco^+^ exosome pathway of epithelial cells to regulate the activity of immune cells, especially to activate B cells with inhibitory activity, which suppresses immunity by activating macrophages by increasing IL-10 levels (Yang et al., 2012). AMs can secretes extracellular vesicles containing suppressor of cytokine signaling (SOCS) protein (Bourdonnay et al., 2015). Mycoplasma bovis infection increases the expression of SOCS(Mulongo et al., 2014). Extracellular vesicles containing SOCS protein are taken up by AECs, downregulate cytokine-induced STAT activation, maintain AECs at rest (Speth et al., 2019), and inhibit the production of type 2 cytokines by AECs to reduce allergic airway inflammation (Draijer et al., 2020).

**Figure 3:**
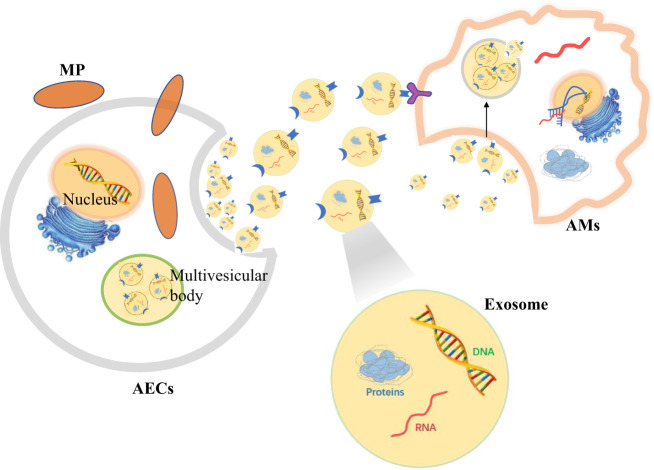
Alveolar epithelial cells (AECs) contact alveolar macrophages (AMs) to resist *Mycoplasma pneumoniae* (MP) by secreting exosomes.

Therefore, when AECs are stimulated by MP, signals can be transmitted to AMs by secreting extracellular vesicles to regulate the immune response *in vivo* (**Figure 3**).

## Establishment of gap junction between AMS and AECS during MP infection

Gap junction, which is composed of connexins (Cx), is an intercellular channel that allows molecular communication between adjacent cells (Trosko, 2001). Such changes in communication can cause pathological reactions (Trosko and Chang, 1986; Trosko et al., 1992; Spath et al., 2013). Gap junction channels allow the transfer of ions and small compounds between cells, so as to coordinate the multiple metabolic and electrical functions of cell communities (Bruzzone et al., 1996; Eugenin et al., 1998). There are 21 connexins isomers in the human proteome that have different physiological properties and regulatory responses. Some are expressed in single-cell types and form heteromeric (more than two different connexins in a connexon) or heterotypic (A Gap junction channel with different Connexons) channels, thus further diversifying its composition and function (Maeda and Tsukihara, 2011). Many clinical studies have shown that the gap junction of connexin 43 (Cx43) and connexin 40 (Cx40) is related to non-infectious lung inflammation (Ni et al., 2017; Ni et al., 2018; Zhang HC et al., 2018).

Gap junctions are usually intercellular contacts between AECs, but also exist between AMs and AECs (Bissonnette et al., 2020). Gap junctional communications between members of a cell community allow the coordination of cellular, metabolic, and electrical responses (Riches et al., 1988; Eugenin et al., 1998). Westphalen et al. first discovered that AMs attached to the alveolar wall form gap junction channels containing connexin 43 (Cx43) with epithelial cells through real-time *in situ* imaging of mouse alveoli in 2014. During LPS induced inflammation, AMs attach to alveoli to form gap junction channels. It established mutual communications between AMs and epithelial cells through synchronized Ca2^+^-dependent Akt activation. Cx43 deletion in AMs increases the secretion of cytokines that may be mainly derived from AMs (MIP-1α) and AECs (CXCL1). Cx43 deletion also causes an increase in neutrophil recruitment. This suggests that AMs and AECs can mutually inhibit the release of cytokines (Westphalen et al., 2014). Cx43 gap junction between macrophages and epithelial cells has also been found in human cells (Oumouna et al., 2015; Beckmann et al., 2020), suggesting that it is involved in the communication of human lung cells. However, the exact role of the communication about gap junction channels remains uncertain. Therefore, when the pathogen invades one or more sensitive cells in the cluster, all cells in the cluster communicate with each other through Ca2^+^-dependent gap junctions and transmit signals to other types of cell populations.

It is well known that MP causes a significant increase in pro-inflammatory factors TNF-α and IL-1β (Liu et al., 2018; Maunsell and Chase, 2019; Luo et al., 2021). As early as 2003, TNF- α has been shown to increase the expression, adhesion and extravasation of Cx43 in monocytes/macrophages (Eugenín et al., 2003; Véliz et al., 2008). Research shows that TNF-α alone does not increase Cx43 total protein levels in mouse or human dendritic cells, but in combination with IL-1β can enhance the expression of functional Gap junctions between cultured dendritic cells (Corvalan et al., 2007; Mendoza-Naranjo et al., 2007). Therefore, after MP infection, an increase in secretion of TNF-α and IL-1β can increase the expression and adhesion of Cx43 in macrophages, form Cx43 gap junction channels between AMs and AECs, and transmit immunosuppressive signals through Ca^2+^ waves.

## SP-A-linked communications between AMS and AECS during MP infection

As mentioned earlier, a strong inflammatory response can cause lung injury. Studies have shown that pulmonary surfactant protein A (SP-A) inhibits local inflammatory response by inhibiting the synthesis and release of cytokines and inflammatory mediators (Bates, 2010; Younis et al., 2020). Pulmonary surfactant-associated protein (SP) is a lipoprotein complex synthesized and secreted by AECIIs and airway Clara cells. It is an important component of pulmonary surfactant. SP proteins usually contain a N-terminal collagen like domain and a C-terminal protein recognition domain (CRD). In the Ca2^+^-dependent response, CRD binds a variety of ligands, including a variety of immune cell surface receptors and pathogen-derived carbohydrates, which enhances the phagocytosis of pathogens and participates in the regulation of immune cell function and immune inflammatory response (LeVine et al., 1999a; LeVine et al., 1999b; Sano and Kuroki, 2005). The content of SP-A is the most abundant, accounting for about 50% of the total SP. The change of SP-A content is closely related to intrapulmonary and extrapulmonary diseases (Ujma et al., 2017; Vieira et al., 2017).

The presence of a specific receptor for SP-A on the surface of AMs was found as early as 1992,so SP-A from AECIIs can bind specifically to rat alveolar macrophages (Pison et al., 1992). SP-A amplifies IL-4-mediated phosphorylation of STAT6 and Akt by binding to myosin18A in AMs to increase AMs proliferation and alternative activation (Garcıa-Fojeda et al., 2022). SP-A not only plays a role in regulating AMs cycle, but also in regulating AMs inflammation. SP-A can up-regulate activity of the mannose receptor and promote AMs to phagocytize pathogens and apoptotic neutrophils, promoting the regression of alveolar inflammation (Schagat et al., 2001; Reidy and Wright, 2003). The mannose receptor, a pattern recognition receptor expressed on macrophages, can recognize and bind specific sugar molecules through extracellular regions, which play a role in recognizing pathogens, presenting antigens and maintaining internal environmental stability. (Beharka et al., 2002). The study also found that the CRD domains of SP-A bind to the soluble extracellular domains of TLR2, TLR4 and MD-2 receptors *via* a Ca^2+^-dependent pathway (Awasthi et al., 2019; Ghaffarpour et al., 2020), and SP-A can regulate the expression of TLR2 and TLR4 in macrophages after transcription (Que and Shen, 2016). However, although SP-A can up-regulate the expression of TLR2 in macrophages, it also inhibits the TLR2-mediated NF-κB signaling pathway. SP-A affects NF-κB signaling by affecting key regulatory factors in the signaling pathway, such as the phosphorylation of IκBα, the heterotopia of p65, the phosphorylation of MAPK family members and the phosphorylation of Akt, resulting in a significant decrease of TNF-α and inhibiting the excessive inflammatory response induced by pathogen stimulation (Nayak et al., 2012; Nguyen et al., 2012).

Mycoplasma express several cell surface ligands that can interact with SP-A. It contains a class of high affinity ligands of SP-A composed of unsaturated phosphatidylglycerols (Piboonpocanun et al., 2005). Kannan et al. also identified a specific membrane protein MPN372, which also has high affinity with SP-A (Kannan et al., 2005). Therefore, SP-A protein on the surface of AECIIs can recognize and bind MP. Moreover, studies have shown that SP-A could clear MP after macrophages recognizing it. In the absence of SP-A, CFU of MP in host cells is not significantly reduced after mycoplasma infection of IFN-γ-activated macrophages (Hickman-Davis et al, 1998). The variation of SP-A gene on the surface of AECIIs has the same effect as the deletion of SP-A gene, which will significantly enhance the binding with MP membrane protein and promote the host inflammatory response (Ledford et al., 2015). However, in MP infected the mouse model, the expression levels of pro-inflammatory factors such as B7-H3 and IL-13 were up-regulated, but the expression level of SP-A was significantly reduced (Hao et al., 2022). Therefore, the SP-A protein secreted by AECIIs is related to the inhibition of excessive inflammatory response of AMs stimulated by MP. However, MP will inhibit the secretion of SP-A by AECIIs.

**Figure 4:**
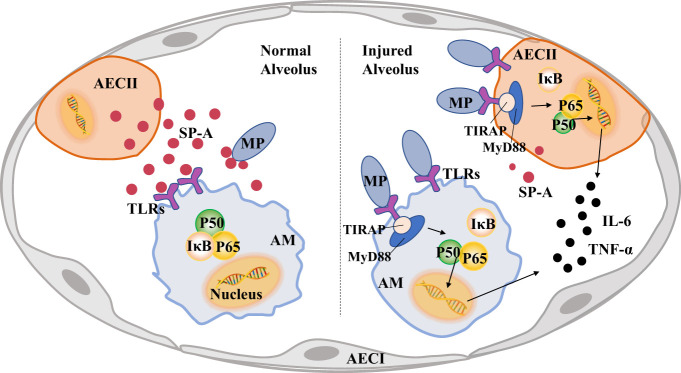
Pulmonary surfactant protein A (SP-A) links alveolar macrophages (AMs) and alveolar epithelial cells (AECs) to reduce lung injury caused by *Mycoplasma pneumoniae* (MP). A large amount of MP will activate TLRs-MyD88-NF-κB of AECs signaling pathway, increase the secretion of inflammatory factors and reduce the production of SP-A, MP also activates the pro-inflammatory phenotype of AMs and causes lung injury.

As shown in **Figure 4**, after MP invades the alveoli, AMs activate immune pathways to cause pulmonary inflammatory response and eliminate MP. SP-A secreted by AECIIs can recognize and bind MP, and bind to TLRs of AMs at the same time. It can reduce the lung injury caused by MP by inhibiting NF-κB and other signal pathways in AMs. However, the invasion of MP into the lung will reduce the secretion of SP-A in AECIIs and induce pulmonary inflammation.

## Discussion

This review mainly summarizes the possible pathways of information transmission between AMs and AECs after MP enters the lung. The process of interaction between AMs and AECs against MP can be divided into promoting beneficial inflammatory response and inhibiting excessive inflammatory response.

In the process of the host against MP, the communication between AECs and AMs removes the MP by promoting inflammation. After MP is recognized by TLR on the surface of epithelial cells, it activates the signaling pathways such as MyD88-NF-κB to secrete pro-inflammatory cytokines and exosomes carrying miRNA and other pro-inflammatory substances. These products can transmit signals to AMs and activate the pro-inflammatory response of AMs. In addition, MP can inhibit the release of IL-10 and other anti-inflammatory factors by the AECs, so that the AMs maintain a pro-inflammatory phenotype. The wandering AMs also recognize MP through surface TLRs and secrete IL-1, IL-23 and other inflammatory factors, which directly or indirectly communicate with the AECs to magnify the pulmonary inflammatory response to eliminate MP.

But persistent inflammation can cause damage to the body, so it is also important that AMs communicate with AECs to suppress the excessive inflammation caused by MP. When MP is infected, AECs and AMs secretes TGF-β, IGF-1 and other cytokines to inhibit inflammatory responses and to avoid excessive damage caused by inflammation by regulating PI3K/AKT and other signaling pathways. AECs and AMs also transmit immunosuppressive signals by forming protein gap junction such as Cx43 and secreting extracellular vesicles containing anti-inflammatory mediators. SP-A from AECs will emit immunosuppressive signal after binding with MP membrane protein to suppress the excessive immune response induced by MP. When SP binds with TLRs of AMs, it will transmit immunosuppressive signal to AMs, and then regulate MyD88-NF-κB and other signaling pathways to weaken inflammatory response.

On a final note, there are still many questions that need further study. For example, SP-A can regulate the expression of TLR2 and TLR4 in AMs and enhance MP phagocytosis by AM(Schagat et al., 2001; Reidy and Wright, 2003), but how SP-A regulates MyD88-NF-κB in AMs to inhibit inflammatory response is not clear. The mechanism of SP-A from AECIIs regulating AMs needs to be further explored.

In conclusion, when MP invades the lung, signal communications between AECs and AMs play an important role in clearing pathogens and maintaining pulmonary homeostasis.

## Author contributions

HH contributed to the conception of the study. YX dealt with the case and wrote original draft. MW and HH helped modify the content of the article. All authors contributed to the article and approved the submitted version.
